# In Vitro and In Vivo Comparison of Lymphocytes Transduced with a Human CD16 or with a Chimeric Antigen Receptor Reveals Potential Off-Target Interactions due to the IgG2 CH2-CH3 CAR-Spacer

**DOI:** 10.1155/2015/482089

**Published:** 2015-11-17

**Authors:** Béatrice Clémenceau, Sandrine Valsesia-Wittmann, Anne-Catherine Jallas, Régine Vivien, Raphaël Rousseau, Aurélien Marabelle, Christophe Caux, Henri Vié

**Affiliations:** ^1^UMR INSERM U892, 8 Quai Moncousu, 44007 Nantes Cedex, France; ^2^Centre Hospitalier Universitaire de Nantes, 1 Place Ricordeau, 44000 Nantes, France; ^3^UMR INSERM 1052, Centre Léon Bérard, 28 rue Laennec, 69008 Lyon, France; ^4^Genenthec Inc., South San Francisco, CA 94080, USA

## Abstract

The present work was designed to compare two mechanisms of cellular recognition based on Ab specificity: firstly, when the anti-HER2 mAb trastuzumab bridges target cells and cytotoxic lymphocytes armed with a Fc receptor (ADCC) and, secondly, when HER2 positive target cells are directly recognized by cytotoxic lymphocytes armed with a chimeric antigen receptor (CAR). To compare these two mechanisms, we used the same cellular effector (NK-92) and the same signaling domain (Fc*ε*RI*γ*). The NK-92 cytotoxic cell line was transfected with either a Fc*γ*RIIIa-Fc*ε*RI*γ* (NK-92^CD16^) or a trastuzumab-based scFv-Fc*ε*RI*γ* chimeric receptor (NK-92^CAR^). In vitro, the cytotoxic activity against HER2 positive target cells after indirect recognition by NK-92^CD16^ was always inferior to that observed after direct recognition by NK-92^CAR^. In contrast, and somehow unexpectedly, in vivo, adoptive transfer of NK-92^CD16^ + trastuzumab but not of NK-92^CAR^ induced tumor regression. Analysis of the in vivo xenogeneic system suggested that the human CH2-CH3 IgG2 used as a spacer in our construct was able to interact with the FcR present at the cell surface of the few NSG-FcR+ remaining immune cells. This interaction, leading to blockage of the NK-92^CAR^ in the periphery of the engrafted tumor cells, stresses the critical role of the composition of the spacer domain.

## 1. Introduction

The clinical benefits associated with adoptive immunotherapy of some mAbs have established the clinical pertinence of several antigens as immune therapeutic targets. For some therapeutic antibodies such as the anti-CD20 rituximab or the anti-HER2 trastuzumab, cell-mediated immunity (antibody-dependent cellular cytotoxicity (ADCC)) has been recognized as one of the mechanisms responsible for their clinical efficiency [1, 2]. Accordingly, several strategies have been considered to increase the ADCC potential in patients [3]. Cellular subsets capable of mediating ADCC include neutrophils, monocytes/macrophages, and a subset of natural killer (NK) cells. As an example of a strategy to improve patient ADCC potential, we have shown in the context of breast cancer patients that their NK population could be amplified in vitro up to 425-fold using a straightforward culture procedure [4]. Yet, because of technical limitations associated with the clinical use of NK cells (poor recovery after freezing and thawing, poor in vitro expansion compared to T cells) and the numerous approaches that have become available to engineer lymphocytes (see for review [5]), we have also considered the possibility of arming T cells with a receptor that would enable them to mediate ADCC [3]. In the above study, we have shown that, after transduction with Fc*γ*RIIIa/Fc*ε*RI*γ* (referred to as CD16/*γ*) receptor fusion gene, CD4+ and CD8+ cytotoxic T lymphocytes displayed a stable expression of the CD16/*γ* receptor at their surface and mediated ADCC. Thus, associating a therapeutic mAb and an adoptive transfer of CD16/*γ* transduced T cells could combine the advantages associated with the functional potential of cytotoxic lymphocytes and recognition of the target cells unrestricted by the major histocompatibility complex. Another way to reach the same objective is to redirect T cells with the so-called chimeric T cell receptor (CAR) or “T bodies,” a strategy pioneered more than two decades ago by the team of Eshhar [6–8], which has recently shown impressive antitumor effects in patients with hematologic diseases (for a review see Gill and June [9]). These are fusion proteins between single chain variable fragments (scFv) from a monoclonal antibody and an intracellular signaling domain such as CD3*ζ* or Fc*ε*RI*γ*. The above two strategies will be referred to in the text as the ADCC approach (a treatment relying on an mAb + an adoptive transfer of CD16 armed T cells) and the CAR approach (the adoptive transfer of CAR armed T cells). Both strategies have the same fundamental advantage, which is the combination of the cellular immune potential of T cells with recognition of a target cell unrestricted by the MHC. But they also differ on several clinical, immunological, and practical aspects. In particular, in terms of safety, as highlighted by Morgan et al. in their in-depth analysis of a serious adverse event following the administration of T cells transduced with a CAR against HER2 [10], a major difference between mAb administration and adoptive transfer of CAR engineered T cells is that while Ab is cleared by the body, CAR T cells can continuously produce effector cytokines and can expand in cell numbers following antigen stimulation. This may be a real advantage or a real danger, depending on the tumor restriction of the antigen targeted. Provided that the tumor antigen targeted is appropriately restricted, the main advantage of the CAR approach would be its efficiency. On the other hand, the ADCC approach allows the search of a correct dosage simply by changing the dose of the mAb. The aim of the present study was to directly compare the CAR and ADCC approaches with a clinically relevant target antigen. To this end, as a first step in designing a model, we equipped the same cytotoxic lymphocyte line (the human NK cell line NK-92) with either a Fc*γ*RIIIa/Fc*ε*RI*γ* receptor (referred to as NK-92^CD16^) or an anti-HER2/Fc*ε*RI*γ* CAR receptor (referred to as NK-92^CAR^) and compared their efficiency in killing HER2 positive tumor target cells in vitro and in vivo. While in vitro comparison between these two effectors has highlighted an advantage in the CAR approach in terms of cytotoxic potency, the in vivo experiments performed in NSG mice have not enabled a CAR/ADCC comparison but instead have revealed an off-target interaction which blocked the potential antitumoral efficacy of the CAR modified lymphocytes.

## 2. Results

### 2.1. Expression of the Chimeric Anti-HER2 Receptor (CAR) and the CD16 on the Surface of NK-92

**Figure 1:**
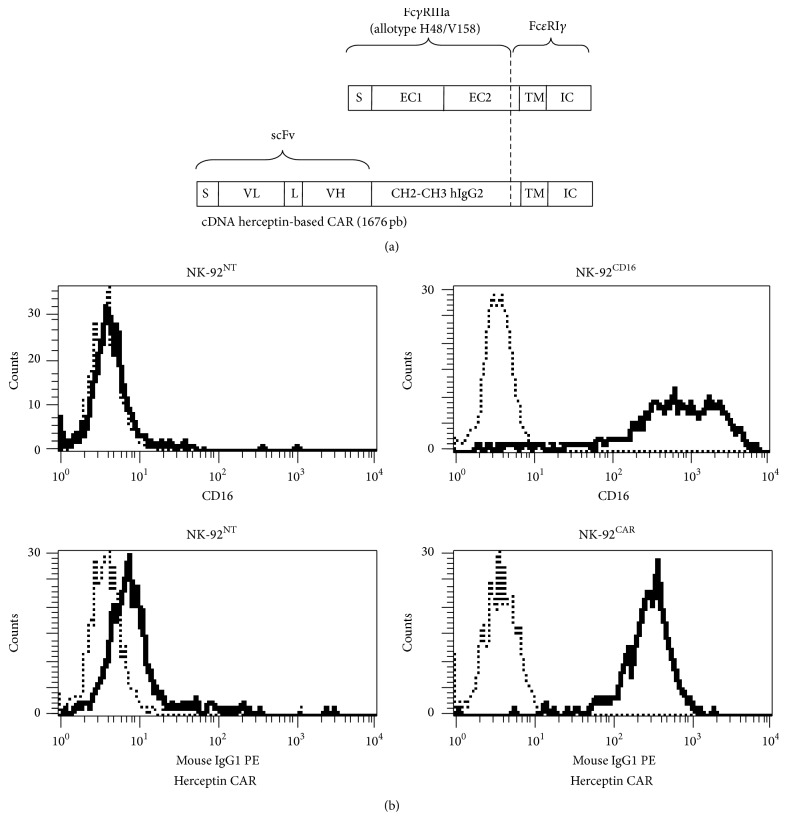
CD16 and CAR vector design and expression in NK-92 cells. (a) Schematic representation of the chimeric human Fc*γ*RIII-human Fc*ε*RI*γ* molecule and the trastuzumab- (4D5-) based CAR against HER2: the CD16/*γ* chimeric cDNA comprised the leader (S) and the two extracellular domains (EC1 and EC2) of human CD16^H48V158^, two amino acids (aa) of the extracellular domain of human Fc*ε*RI*γ*, and the intact transmembrane (TM) and intracellular (IC) domains. The trastuzumab- (4D5-) based CAR contains the VL: hum Ab 4D5-8 light chain; a linker; VH: hum Ab 4D5-8 heavy chain and two amino acids (aa) of the extracellular domain of the human Fc*ε*RI*γ*; and the intact transmembrane (TM) and intracellular (IC) domains. (b) Transgene expression of NK-92 cell line transduced with CD16-*γ* chimeric receptor or trastuzumab-*γ* CAR. Anti-CD16 (clone 3G8) was used to determine CD16 expression on CD16-*γ* transduced NK-92 cell line (solid line), and an isotype Ab was used as negative control (dotted line). Anti-human IgG2a-Fc (clone HP6002) was used to determine trastuzumab-*γ* CAR expression on trastuzumab-*γ* CAR transduced NK-92 cell line (solid line), and an isotype Ab was used as negative control (dotted line).

The chimeric cDNAs were synthesized by GeneCust (Dudelange, Luxembourg). The CD16/*γ* chimeric cDNA comprises the leader (S) and the two extracellular domains (EC1 and EC2) of human CD16^H48V158^ and two amino acids (aa) of the extracellular domain of the human Fc*ε*RI*γ* (Pro4-Gln5), as well as the intact transmembrane (TM) and intracellular (IC) domains (Figure 1(a)). The trastuzumab-based CAR contains the VL and VH from the mAb (Ab4D5-8), separated by a linker, the human CH2-CH3 IgG2 as a spacer, and the same signaling domain as that of CD16 (Figure 1(a)). After transduction (see Section 4), 41% of the NK-92 expressed CD16 and 36% expressed the CAR. After immunomagnetic purification using anti-CD16 and anti-human IgG2a-Fc-specific mAb (see Section 4), essentially pure populations of NK-92^CD16^ and NK-92^CAR^ were obtained (Figure 1(b)).

### 2.2. In Vitro Comparison of HER2-Specific Cytotoxicity Mediated by NK-92^CD16^ + Trastuzumab or NK-92^CAR^

**Figure 2:**
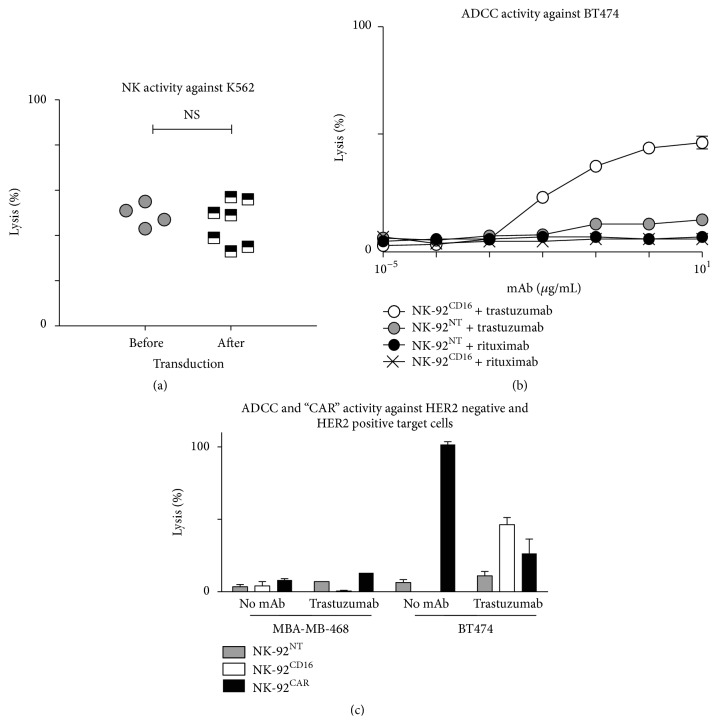
Cytotoxic activity of NK-92^CD16^ and NK-92^CAR^. (a) Spontaneous (NK) activity of NK-92 before and after transduction (E/T ratio: 30/1). (b) The effector cells NK-92^CD16^ and NK-92^CAR^ were tested against the HER2 positive BT474 cell line preincubated in the presence of increasing concentration of trastuzumab (mean of two experiments). (c) Cytotoxic activity of NK-92^NT^, NK-92^CD16^, and NK-92^CAR^ and against HER2 negative (MBA-MB-468) or HER2 positive (BT474) cell lines (trastuzumab, 10 mg/mL, E/T ratio: 30/1). Cytotoxicity was evaluated from ^51^Cr release after 4 hours of incubation. The data represent the means from three independent experiments.

**Figure 3:**
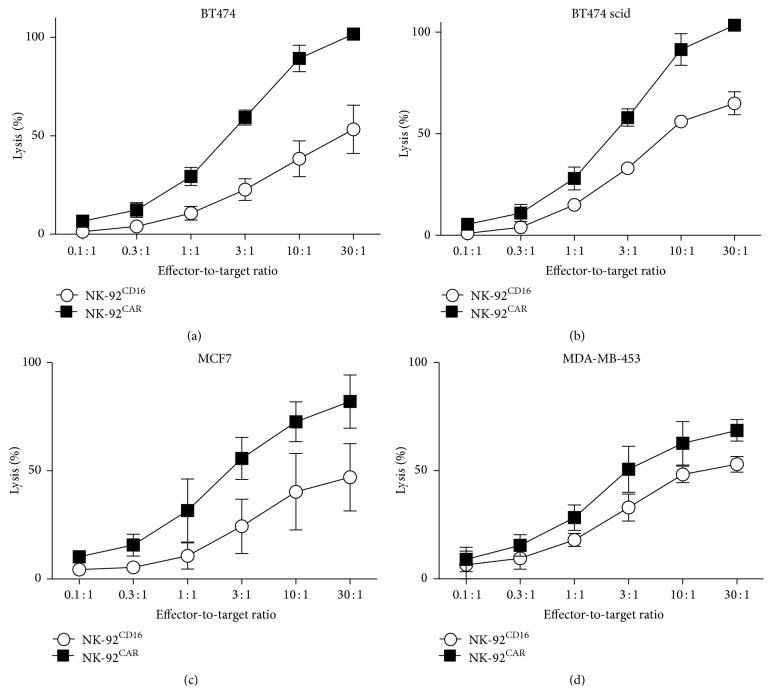
Comparison of direct NK-92^CAR^ or indirect NK-92^CD16^ recognition of 4 HER2 positive cell lines. Cytotoxicity was evaluated from ^51^Cr release after 4 hours of incubation; the data represent the means of three independent experiments. For ADCC, cell lines were incubated in the presence of 10 *μ*g/mL trastuzumab. In the absence of antibody BT474, MDA-MB-453 and MCF7 were not sensitive to NK activity of NK-92 (see Figure 2 and data are not shown for MCF7).

First, we showed that the spontaneous cytotoxic activity (tested against K562, E/T ratio: 30/1) of the untransduced and the transduced NK-92 was not significantly modified by the retroviral transduction (Figure 2(a)). Next, ADCC activity of the NK-92^CD16^ against the BT474 cell line was tested in the presence of increasing concentration of trastuzumab (as in the example shown in Figure 2(b)) and demonstrated a plateau (close to 50% specific lysis) at 10 *μ*g/mL (E/T ratio: 30/1). No ADCC was observed in the presence of rituximab and NK-92^CD16^ or NK-92^NT^. Note that a background lysis was observed for NK-92^NT^ + trastuzumab (compared to NK-92^NT^ + rituximab), likely due to background expression of CD16 by the NK-92 cell line. For all other in vitro experiments, trastuzumab was used at a concentration of 10 *μ*g/mL. Next, we compared the efficiency of target cell lyses induced either after direct recognition of the HER2 Ag by the NK-92^CAR^ alone or after indirect recognition by the NK-92^CD16^ in the presence of trastuzumab. To this end, target cells (the HER2 negative MDA-MB-468 and the HER2 positive BT474) were coated or not with trastuzumab, and the untransduced NK-92^NT^ was used as a negative control. Cytotoxic activities of NK-92^CAR^ and NK-92^CD16^ against trastuzumab coated or not coated MDA-MB-468 and BT474 are summarized in Figure 2(c) (at an effector-to-target ratio of 30 : 1). Neither NK-92^CAR^ nor NK-92^CD16^ presented a significant level of cytotoxicity against the HER2 negative MDA-MB-468 cell line (Figure 2(c), left panel). When tested against the BT474, NK-92^CAR^ showed a high level of cytotoxic activity (101 ± 3% at 30 : 1). Killing of BT474 was also observed by NK-92^CD16^ in the presence of trastuzumab, although this occurred at a level below that observed by NK-92^CAR^ (46 ± 8% at the same effector-to-target ratio of 30 : 1). In addition, the preincubation of the BT474 in the presence of trastuzumab drastically reduced the killing by NK-92^CAR^ (Figure 2(c), right panel). To confirm the differences in cytotoxic performance between NK-92^CD16^ and NK-92^CAR^, further comparison was performed against 4 different HER2 positive cell lines: BT474, BT474 scid (a subclone of BT474 used for the in vivo experiments), MCF7, and MDA-MB-231 (Figure 3). These data showed that, in vitro, in these experimental conditions, based upon 4 hr cytotoxicity assays, the direct pathway of killing (by NK-92^CAR^) was always more efficient than the indirect pathway (by NK-92^CD16^) (Figures 3(a), 3(b), 3(c), and 3(d)).

### 2.3. In Vivo Comparison of NK-92^CD16^ + Trastuzumab and NK-92^CAR^ to Control the Growth of Established BT474 Tumor

**Figure 4:**
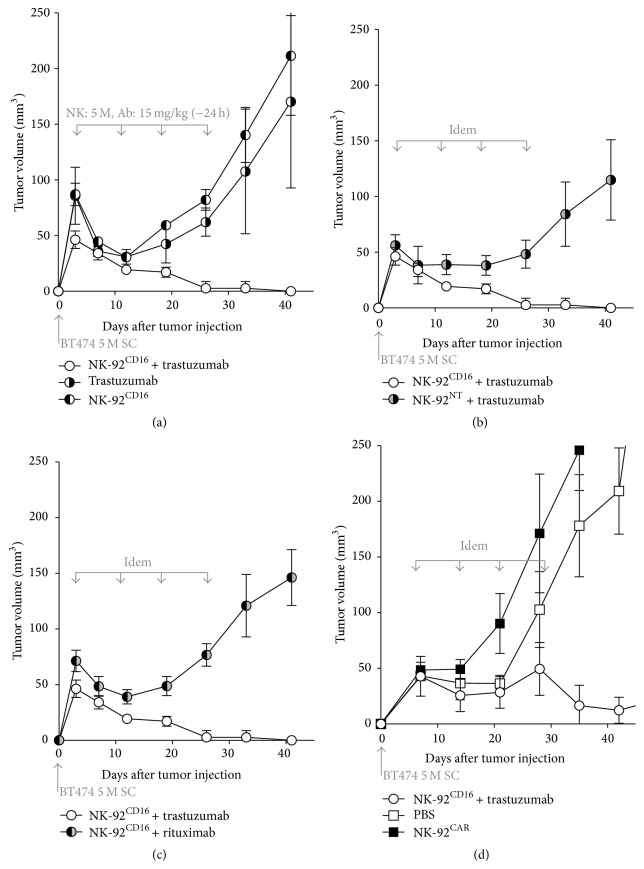
The NK-92^CD16^ in the presence of trastuzumab but not NK-92^CAR^ induces regression of the HER2 positive BT474 tumor engrafted in NSG mice. Results presented in (a), (b), and (c) are from the same series of experiments but are presented separately for readability: the group of mice treated with NK-92^CD16^ + trastuzumab is thus the same for (a), (b), (c), and (d). Six-week-old female NSG mice were injected subcutaneously in the left side with 5 × 10^6^ BT474 cells. When tumors reached a minimal volume of 50 mm^3^ (within 5–7 days), mice were individually identified and randomly assigned to the control or treated groups (5 to 15 mice per group) and the indicated treatments were initiated.

Having shown that NK-92^CAR^ were more effective in vitro than NK-92^CD16^ against several HER2 positive tumor cells, we developed a mouse xenograft model to test whether such difference could also be evidenced in vivo. Six-week-old female NSG mice were injected subcutaneously in the left side with 5 × 10^6^ BT474 cells, and when the tumor reached a volume of 50 mm^3^, the mice received the indicated treatment (all mice were sacrificed before the tumor volume reached 2500 mm^3^). We first tested the capacity of IP injection of NK-92^CD16^ and trastuzumab to control the growth of a subcutaneous established NK-92 resistant BT474 carcinoma (Figure 4(a)). In the absence of addition of effector cells, weekly injections of trastuzumab (15 mg/kg) alone do not prevent BT474 tumor burden (Figure 4(a)). When 5 × 10^6^ NK-92^CD16^ cells were used in combination with trastuzumab (15 mg/kg) injected 24 h prior to NK cells, complete tumor regression was observed. Note that tumor regression began after a single dose but that complete regression required 4 injections once per week of 5 × 10^6^ NK-92^CD16^ cells (Figure 4(a)). To confirm that ADCC was the mechanism responsible for tumor regression, experiments were repeated with the untransduced NK-92^NT^ or with NK-92^CD16^ in the presence of an irrelevant antibody (rituximab, directed against CD20, an Ag not present at the surface of BT474). As the result of a mechanism of tumor destruction due to ADCC, neither the untransduced NK-92^NT^ in the presence of trastuzumab nor NK-92^CD16^ in the presence of rituximab were able to control the growth of the BT474 tumor (Figures 4(b) and 4(c), resp.).

We then analyzed the antitumor effect of NK-92^CAR^ in this BT474 xenograft model. In contrast to the in vitro situation, where NK-92^CAR^ has demonstrated a higher efficacy of BT474 killing compared to NK-92^CD16^ + trastuzumab, no tumor regression was observed even after 4 injections with NK-92^CAR^ (Figure 4(d)). Before investigating in more detail the possible reasons for the discrepancy between in vitro and in vivo results, we first controlled the injection route and reproduced the experiment. With NK-92^CAR^ IV instead of IP injection, we observed the same inefficacy (data not shown).

### 2.4. NK-92^CAR^ Scattered into All Organs but Did Not Spread within the Tumor

**Figure 5:**
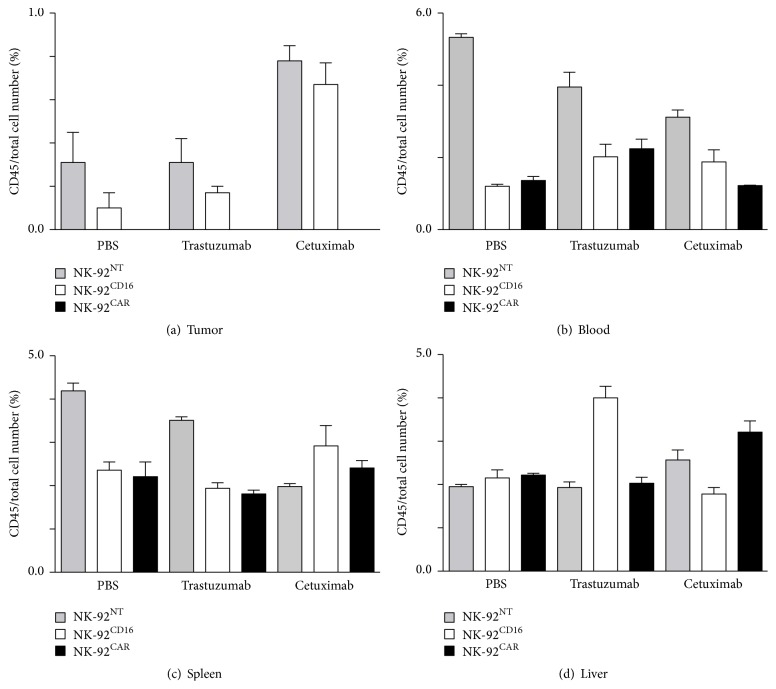
Tissue distribution of NK-92^NT^, NK-92^CD16^, and NK-92^CAR^ in NSG mice bearing an established BT474 tumor. NK-92^NT^, NK-92^CAR^, or NK-92^CD16^ CSFE labelled cells (20 × 10^6^) were injected into NSG mice bearing BT474 tumors (tumor size range of 50 to 100 mm^3^). Twenty-four hours later, mouse blood, liver, tumor, lung, and spleen were collected, and the percentages of CD45 positive cells were analyzed by FACS analysis (percentage of CD45 was calculated from 50,000 events, and the means of two independent experiments are presented).

**Figure 6:**
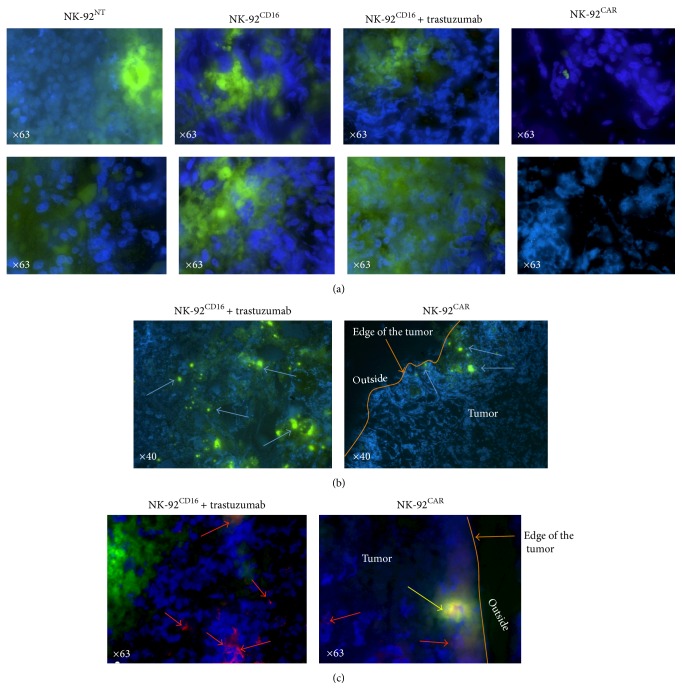
Immunohistochemical analysis of BT474 tumor sections. NK-92 were directly stained with CSFE and tumor cells were visualized by counterstaining with DAPI. (a) NK-92^NT^ and NK-92^CD16^ could be found in different parts of the tumors, either as a cluster or as single cells and preinjection of trastuzumab did not affect NK-92^CD16^ frequency within the tumor (panels 2 and 3). Panel 4 shows the absence of NK-92^CAR^ within the tumors. (b) Comparison of effectors spreading between NK-92^CD16^ and NK-92^CAR^ within the tumors using direct CFSE visualisation (green): NK-92^CAR^ appeared clustered at the edge of the tumor only whereas NK-92^CD16^ are observed everywhere in the tumor. (c) Merge colocalization (yellow arrow) of mice macrophages (red) using Iba1 staining and NK-92^CAR^ cells (green) are always located at the edge of the tumor (blue) whereas isolated macrophages (red arrow) can be found everywhere within the tumor. This was not observed with NK-92^CD16^. Typical representative images are presented.

To investigate the unexpected total lack of in vivo efficacy of NK-92^CAR^, we compared the dissemination abilities of NK-92^CAR^ and NK-92^CD16^ in the NSG mouse model. For this purpose, 2 × 10^7^ NK-92^NT^, NK-92^CAR^, or NK-92^CD16^ cells were injected into NSG mice bearing BT474 tumors (tumor size ranging between 50 and 100 mm^3^). Twenty-four hours later, mouse blood, liver, tumor, lung, and spleen were collected, and the percentages of CD45 positive cells were analyzed by FACS analysis using specific Ab. As shown in Figures 5(b), 5(c), and 5(d), no major differences between NK-92^NT^, NK-92^CAR^, and NK-92^CD16^ spreading were found within blood, spleen, and liver, suggesting no major differences in their ability to recirculate within the NSG mice after IP injection. However, whereas NK-92^NT^ and NK-92^CD16^ can be found in tumors even at a low but significant ratio, no NK-92^CAR^ could be found inside the tumor (Figure 5(a)). We then analyzed whether preinjection of either trastuzumab, competing for HER2 binding with CAR-HER2, or Cetuximab, recognizing HER1 also expressed at the surface of the BT474 cells without interfering with CAR-HER2 binding, could induce a chemoattractive signal for NK cells inside the tumor. As shown in Figure 5(a), opsonization of tumors with specific Ab does not attract NK-92^CAR^ effectors. To confirm these data, we injected 3 × 10^7^ CSFE labelled NK-92^NT^, NK-92^CAR^, or NK-92^CD16^ cells into NSG mice bearing BT474 tumors (tumor size ranging between 50 and 100 mm^3^) and collected the tumors 72 hours later. We then performed IHC on frozen sections by direct reading of CFSE labelled NK effectors and visualization of the tumor cell nuclei by counterstaining with DAPI. As shown in Figure 6(a), NK-92^NT^ and NK-92^CD16^ could be found in different parts of the tumors, either as a cluster or as single cells. Preinjection of trastuzumab did not affect the NK-92^CD16^ frequency within the tumor (Figure 6(a), panels 2 and 3). In support of the previous FACS analysis, NK-92^CAR^ effectors could never be found within the tumors. We observed that where present at all, most NK-92^CAR^ were blocked at the edge of the tumor in the form of clusters (Figure 6(b), right panel) whereas NK-92^CD16^ could be found everywhere inside the tumor (Figure 6(b), left panel). Suspecting a possible interaction between mice macrophages and NK-92^CAR^, we counterstained our frozen IHC sections with anti-Iba1 antibodies specific to mouse macrophages (Wako). As shown in Figure 6(c) right panel, a yellow merge staining always located at the edge of the tumor with NK-92^CAR^ was observed, suggesting that mice macrophages (in red) could aggregate with NK-92^CAR^ effectors (in green). This was never observed inside the tumor, or with NK-92^CD16^ for which isolated macrophages are distributed everywhere inside the tumor (Figure 6(c), left panel, red arrow). Taken together, these data suggest that mice macrophages aggregating NK-92^CAR^ might have prevented them from infiltrating inside the tumor, explaining the lack of efficacy for these type of effectors.

### 2.5. In Vitro Specific Interaction between NK-92^CAR^ and FcR^+^ NSG Splenocytes

**Figure 7:**
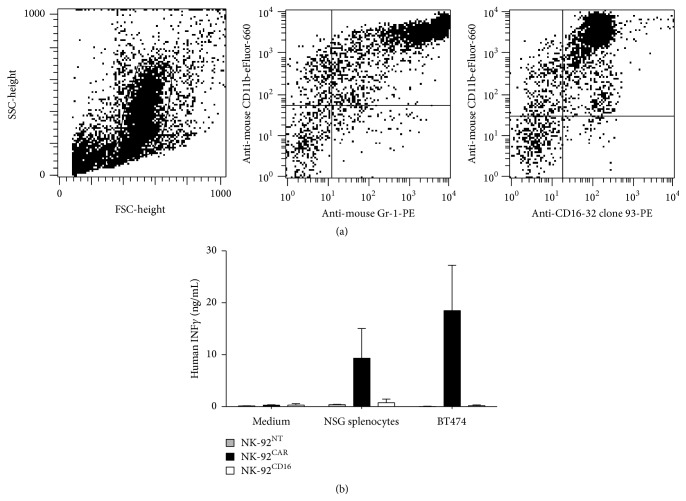
NK-92^CAR^ stimulation after coculture in the presence of NSG splenocytes. (a) Single-cell suspensions from NSG mice spleens were stained with an antibody cocktail and then analyzed by flow cytometry for expression of CD11b (Mac-1) versus Gr-1 (Ly-6G) and CD11b versus CD16/32 (FcR*γ*RIII/FcR*γ*RII). (b) NK-92 (NK-92^NT^, NK-92^CD16^, or NK-92^CAR^) and splenocytes from NSG mice or the HER2 positive cell line BT474 were cocultured at an effector ratio of 5 : 1 and 2 : 1, respectively, in 96-well round bottom culture plates in RPMI 1640 supplemented with 10% FCS and IL2 (100 IU/ML). After 18 h, supernatants were collected and human INF-*γ* content was estimated using an ELISA kit (Affymetrix, e-biosciences). Data represent the mean with SD from three independent experiments.

As shown in Figure 5(a), 24 h after injection, NK-92^NT^ and NK-92^CD16^ are present within the tumor, but not NK-92^CAR^. This was observed whether or not the mice were pretreated with trastuzumab. Because pretreatment of the mice with trastuzumab should have blocked recognition of cross-reactive mice antigens (if any), the interaction between mice macrophages and the CAR was unlikely to be due to the specific VH-VL part of the CAR. In contrast, although NSG mice lack T cells, B cells, and natural killer cells, they nevertheless still have neutrophils, monocyte/macrophages, and dendritic cells. And although these cells are functionally defective because of the NOD/ShiLt genetic background, they nevertheless bear Fc receptors (as shown in Figure 7(a), the CD16-32 staining of the CD11b+/Gr-1+ myeloid NSG splenocytes). And mice Fc receptors can cross-react with the human Fc (see Section 3).

To directly address the possibility of interaction between NSG Fc-bearing cells and the NK-92^CAR^, NSG splenocytes were cocultured with NK-92^NT^, NK-92^CAR^, or NK-92^CD16^ at a 5 : 1 ratio. After 18 hours, NK-92 cell activation was analyzed by quantifying human IFN*γ* in supernatant by ELISA. As shown in Figure 7(b), coincubation of NSG splenocytes with NK-92^NT^, NK-92^CAR^, or NK-92^CD16^ led to detectable production of IFN*γ* only by NK-92^CAR^. This production level is far from being insignificant as it corresponds to half of the IFN*γ* level produced by NK-92^CAR^ after coincubation with the HER2 BT474 tumor cells (9.3 ng/mL and 18.4 ng/mL, resp.). Taken together, these results strongly suggest that NK-92^CAR^ not only have been physically prevented from penetrating the tumor due to being trapped by the surrounding macrophages but also were functionally activated by this off-target interaction.

## 3. Discussion

We compared in the present study two constructs allowing us to implement two strategies relying on an adoptive transfer of cytotoxic lymphocytes to improve the targeting of HER2 positive tumors. In the first, the lymphocytes are equipped with a human CD16 receptor (Fc*γ*RIIIa/Fc*ε*RI*γ* fusion protein) to permit ADCC in the presence of trastuzumab. In the second, we used a first generation CAR encompassing scFv derived from trastuzumab and a CH2-CH3 hIgG2 spacer between the scFv and the transmembrane domain. In both constructs, the signaling domain was the Fc*ε*RI*γ*.

After transduction with CD16 and CAR receptor genes, the human NK cell line NK-92 displayed stable cell surface expression of CD16 and CAR receptors. Specific cytotoxic activity against HER2 positive target cells was demonstrated in both cases, and when we compared their potency against four HER2 positive target cell lines, NK-92^CAR^ always performed better than NK-92^CD16^.

To our knowledge, only two studies have previously compared these two approaches directly. Boissel et al., using the two different therapeutic monoclonal anti-CD20 mAbs rituximab and ofatumumab, demonstrated that the cytotoxic activity of NK-92 cells expressing CD20-targeting first-generation CAR against primary CLL cells was superior to the ADCC by NK-92^CD16^ in the presence of anti-CD20 monoclonal antibodies [11]. And Tassev et al., in their detailed description of the retargeting of NK-92 cells using an HLA-A2-restricted EBNA3C-specific chimeric receptor (a TCR-like antibody), also directly compared the two systems and conclude that “the CAR mediated approach proved far more effective at killing target cells compared with ADCC…” [12].

To what extent can these observations be generalized? For ADCC, the outcome of effector/target cell interactions will depend on the number of CD16 receptors, their affinity with the antibody used to recognize the target, the number of target antigens, nonspecific interactions between the target and effector (e.g., LFA-1/ICAM1), time, concentration of Abs in the liquid phase, and so forth. Similarly for CAR recognition, CAR density, CAR affinity, and antigen expression level will all influence the outcome of the interaction. In addition, for CAR, other variables will also be important, such as the choice of transducing chain, or the addition of an accessory signal (such as the 4-1BB).

In the present study as well as in the study undertaken by Boissel et al. and Tassev et al., because the effector was a clone cell line, the only difference in every effector/target interaction was the way target cells were recognized: either by CD16 + Ab or by the corresponding CAR. Thus, these three studies suggest that the ranking CAR > ADCC remains true (i) against different target antigens (and in particular CD20 and HER2), (ii) with scFv of different affinities (rituximab and ofatumumab have an affinity with the CD20 antigen of approximately 5.45 and 4.76 nM, resp. [13], while the (EBNA clone 315) scFv has an affinity of 291 nM with the HLA-A2/EBNA complex [12]), (iii) with different signaling domains, that is, CD3*ζ* or Fc*ε*RI*γ*, and (iv) with or without the costimulatory signaling domain 4-1BB.

We then attempted to implement a xenogeneic model to continue the comparison in vivo. In NSG mice, we found that IP injection of NK-92^CD16^ and trastuzumab induced total regression of established subcutaneous BT474 tumors (at least until day 40). But in contrast to already published results [14], NK-92^CAR^ that we designed had no antitumor activity, in vivo in NSG. Further analysis demonstrated that, in contrast to NK-92^CD16^, NK-92^CAR^ did not reach the tumor and were aggregated at the periphery of the tumor by the mice macrophages. In addition, in vitro coincubation of NK-92^CAR^ but not NK-92^CD16^ in the presence of NSG splenocytes led to activation and IFN-*γ* production. An important difference between NK-92^CAR^ and NK-92^CD16^, as well as between NK-92^CAR^ used in the present study and that used by Schönfeld et al., was the spacer. For NK-92^CAR^, we used a CH2-CH3 IgG2 hinge, no hinge was used for the NK-92^CD16^, and in their NK-92^CAR^ (also against HER2) Schönfeld et al. used the CD8*α*. Such spacer is sometimes required in the design of a CAR, for the following reasons.

Beyond the variables cited previously, a further specific level of complexity is associated with the use of CAR. The CARs, formerly called “T bodies” by their inventor E. Eschar, can be seen as a sort of TCR having the specificity of an antibody. The TCR recognizes MHC-peptide complexes; these are a unique class of antigens which are characteristic of the exposition at the target cell surface and their physical accessibility to the TCR is homogeneous. The size of a TCR molecule is thus naturally adapted to “sense” the peptide/MHC complex exposed in the target cell. In the same way, the size of the CD16 is naturally adapted to catch the Fc of the Ab bound to the target cell surface, though in this case with more variability than for the TCR, since the exposition of the Fc will depend on the position of the epitope recognized. In nature, this difficulty is solved by the flexibility of the Ab, which may derive from specific characteristics of both the Fab and the Fc region (for a review, see [15]). Consequently, while the steric constraints associated with the target cell recognition by a lymphocyte equipped with a TCR or a CD16 receptor are essentially “natural,” this is not the case for a CAR, whose ability to catch the Ag and transmit the signal will rely in part on the characteristics of the CAR hinge region.

Indeed, the distance of the epitope to the target cell surface, as well as the flexibility and length of the CAR hinge region, matters in regard to CAR recognition. In line with the spatial constraints cited above, it has been shown that a protruding hinge region is needed for efficient activation of lymphocytes armed with a CAR recognizing a membrane-proximal epitope, whereas lymphocytes equipped with a CAR specific for a membrane-distal epitope can be efficiently engaged without extracellular spacer element [16, 17].

As many others before us [17–22], we used an IgG hinge region because at first glance it presented many advantages. First, in line with spatial constraints, the IgG Fc domain can provide both flexibility and the possibility of different lengths by adapting the number of CH2 or CH3 molecules; for clinical applications, it lacks immunogenicity; and for detection, it allows the use of anti-Fc reagents. In addition, we made the choice to use the human IgG2 as a spacer domain in our CAR design because of its lower affinity for the human Fc*γ*Rs [23]. Furthermore, replacement of the IgG1 CH2 sequences with those of IgG2 was shown to eliminate in vitro the activation of CAR T cells by human Fc*γ*R-bearing cells and simultaneous cross-activation of cytokine production by innate immune cells [19].

Clearly, this precaution was not enough. CARs are designed to be used in humans and their study in xenogeneic models may reveal specific traps. In particular, the receptors for the Fc domain of IgG, Fc*γ*Rs, are quite dissimilar in binding abilities and expression pattern between human and mouse [24]. Mice have three activating Fc*γ*Rs (mFc*γ*RI, mFc*γ*RIII, and mFc*γ*RIV) and one inhibitory Fc*γ*RIIb. In NSG mice, neutrophils and monocytes constitute most of the remaining detectable mouse immune cells. Neutrophils express two activating Fc*γ*Rs: Fc*γ*RI and Fc*γ*RIV. Dendritic cells and macrophages are also present in the NSG mouse and although they are functionally defective because of alleles in the NOD/ShiLt genetic background, they express activating Fc*γ*Rs. Dendritic cells express Fc*γ*RI and macrophages express all the Fc*γ*Rs, and even though they are not functional, they could nevertheless interact with the spacer derived from human IgGFc. Moreover, human IgG2 can bind to the murine Fc*γ*RIIb and Fc*γ*RIII and induce a potent ADCC with mouse NK cells and mouse polymorphonuclear leukocytes [25]. Finally, the absence of antitumoral activity of NK-92^CAR^ with the human full-length IgG2 Fc derived spacer observed in the present study supports and extends the results obtained using CD19-CAR designed with a full-length human IgG4 Fc spacer, which failed to eradicate Raji tumors in NSG mice unless the entire CH2 domain responsible for FcR binding was removed [22].

In conclusion, our results extend the number of target antigens for which the CAR approach performs better than the ADCC approach in vitro in terms of cytotoxic activity. Moreover, after the recent work of Hudecek et al. [22], we provide additional evidence stressing the potential dramatic effect in vivo of the spacer domain of CARs, even those devoid of intrinsic signaling capacity.

## 4. Materials and Methods

### 4.1. Cell Lines

NK-92, the human NK cell line (ATCC, Rockville, MD), was grown in RPMI 1640 culture medium (Gibco, Cergy Pontoise, France) supplemented with 10% FBS (PAA Laboratories, Les Mureaux, France), 100 IU/mL IL-2 (Proleukin) (Chiron Corporation, Emeryville, US), 2 mM L-glutamine (Gibco), penicillin (100 IU/mL), and streptomycin (0.1 *μ*g/mL) (Gibco). Epstein-Barr B-lymphoblastoid cell lines (BLCLs) were derived from donor peripheral-blood mononuclear cells (PBMCs) by in vitro infection with EBV-containing culture supernatant from the Marmoset B95-8 cell line (ATCC) in the presence of 1 *μ*g/mL cyclosporin-A. The HER2 negative MDA-MB-468 and the HER2 positive BT-474, MDA-MB-231, and MCF7 breast cancer cell lines were obtained from ATCC. Cell lines were cultured in complete medium consisting of DMEM (Sigma Aldrich, St. Quentin Fallavier, France), 10% heat-inactivated foetal calf serum, 2 mM glutamine (Sigma Aldrich), 100 U/mL penicillin, and 10 *μ*g/mL streptomycin (Sigma Aldrich).

### 4.2. Flow Cytometry

Expression of the trastuzumab- (4D5-) based CAR construction against HER2 and of the human CD16 (Fc*γ*RIIIa/Fc*ε*RI*γ*) on the surface of NK-92 was determined by direct immunofluorescence using anti-CD16 (clone 3G8) and anti-human IgG2a-Fc that recognize the CH1-CH2 spacer of the CAR (clone HP6002), respectively. For staining 0.1 × 10^6^ cells (untransduced NK-92^NT^, NK-92^CD16^, and NK-92^CAR^) were incubated for 15 minutes at room temperature at the indicated mAb concentrations diluted with PBS supplemented with 0.1% human albumin in a final volume of 30 *μ*L. After staining, plates were centrifuged, the supernatant was discarded by flicking, and wells were washed twice with 200 *μ*L ice-cold PBS. Negative controls were set up in the presence of a control isotype in case of direct staining or in the absence of first Abs for indirect staining. In case of indirect staining, cells were washed after the first incubation and the second Ab was used at saturating concentration.

### 4.3. Retroviral Vector Production

Transient retroviral supernatants were produced by CaCl_2_ precipitation with 15 *μ*g of plasmid. Two million Phoenix-Ampho cells [26] were seeded into 10 cm diameter dishes 24 h prior to transfection. The transfection was performed with 15 *μ*g pMX/CD16 or pMX/CAR plasmid DNA using CaCl_2_ precipitation (Invitrogen). The medium (10 mL) was replaced 6 h after transfection. The conditioned medium was collected 48 h after transfection, filtered through 0.45 *μ*m pore-size filters, and kept at −80°C until use. The viral titer was determined by the transduction of Jurkat T cells (1 × 10^6^ cells per well in 6-well plates) with serial dilutions of virus and analyzed for CD16 or CAR expression 4 days after infection. The retroviral supernatant titers were typically 1–5 × 10^5^ IU (Infectious Units)/mL.

### 4.4. NK-92 Cell Line Transduction Using Retroviral Supernatant

The NK-92 cell line was resuspended in RPMI 1640 culture medium supplemented with 10% FBS and 100 IU/mL of recombinant IL-2, seeded at 1 × 10^6^ cells in 1 mL per well into 6-well plates, and exposed to 2 × 2 mL of retroviral supernatant by spinoculation (2400 g, 1.5 h, and 32°C) in the presence of 4 *μ*g/mL polybrene (Sigma, St. Quentin Fallavier, France). The culture medium was changed 24 h after infection. Mock (nontransduced) controls were performed in parallel, by which the supernatant of untransfected packaging cells was added to the NK-92 cell line. The transduction efficiencies were assessed 5 days later by flow cytometry after staining the CD16 and the CAR with a PE-conjugated mouse anti-CD16 (clone 3G8) or a PE-conjugated mouse anti-human IgG2a-Fc (clone HP6002), respectively.

### 4.5. Immunoselection of Transduced NK-92 Cell Lines

After transduction, NK-92^CD16^ and NK-92^CAR^ cells were stained with mouse anti-CD16 (clone 3G8) and anti-human IgG2a-Fc (clone HP6002), respectively, and immunoselected using anti-mouse-IgG coated beads (Dynabeads M-450, Dynal AS, Oslo, Norway), according to the supplier's instructions. Based on CD16 and CAR expression, the purity after immunoselection was >95%.

### 4.6. Cytotoxicity and ADCC Assay

Cytotoxic activity was assessed using a standard ^51^Cr release assay. The target cells were labelled with 100 *μ*Ci ^51^Cr for 1 h at 37°C, washed four times with culture medium, and plated at the indicated effector-to-target cell ratios in 96-well flat-bottom plates. The indicated mAb was incubated with the target cells for 20 mins at room temperature before the addition of effector cells. After 4 h incubation at 37°C, 25 *μ*L of supernatant was removed from each well and mixed with 100 *μ*L scintillation fluid, and ^51^Cr activity was counted in a scintillation counter (MicroBeta, Perkin Elmer, Courtaboeuf, France). Each test was performed in triplicate. The results are expressed as the percentage of lysis, which is calculated according to the following equation: (experimental release − spontaneous release)/(maximal release − spontaneous release) × 100, where the experimental release represents mean counts per minute (cpm) of target cells in the presence of effector cells, spontaneous release represents the mean cpm of the target cells incubated without effector cells, and maximal release represents the mean cpm of the target cells incubated with 1% Triton X-100 (Sigma).

### 4.7. Mice

NOD/SCID-IL2R*γ* ^−/−^ (NSG-JAX) mice were bred and maintained under pathogen-free conditions at the Floralis Jean Roget Institute (UJF Grenoble, France) in sterile intraventilated cages. Mice were acclimated for 1 week before experimental use in Centre Léon Bérard animal facilities (Anican). All animal experiments were performed in compliance with French government guidelines and INSERM standards for experimental animal studies (agreement B69-388-0202). They were approved by the local ethics committee of Centre Léon Bérard, ENS, PBES, and P4 laboratory (CECAPP, Lyon, France). Body weight was monitored weekly and the mice were clinically examined throughout the study.

### 4.8. In Vivo Experimental Model

Six-week-old female NOD/SCID-IL2R*γ* ^−/−^ mice were injected subcutaneously in the left side with 5 × 10^6^ BT474 cells. When tumors reached a minimal volume of 50 mm^3^, mice were individually identified and randomly assigned to the control or treated groups (5 to 15 mice per group) and treatments were initiated. Next, tumor growth was monitored twice a week by measuring two perpendicular diameters with calipers. Tumor volume (*V*) was calculated using the following equation: *V* = (*a* ^2^ × *b*)/2, where *a* is the width of the tumor (small diameter) and *b* the length (large diameter), both in millimeters. Nonirradiated NK-92^NT^, NK-92^CD16^, or NK-92^CAR^ effector cells (5 × 10^6^ each) were injected with IP once a week for 4 weeks. Trastuzumab (15 mg/kg) or rituximab control antibody (15 mg/kg) was given IP 24 hours before injection of NK-92^NT^ or NK-92^CD16^. Mice were sacrificed before the tumor volume reached 2500 mm^3^. Each tumor was dissected and either fixed in formol and processed for histopathological examination or used for RNA extraction. Blood, liver, lung, and spleen from each animal were collected.

### 4.9. Immunohistochemistry

Frozen excision of BT474 tumors was analyzed by immunohistochemistry on 5 *μ*m tissue sections using monoclonal anti-human CD45 (2D1, Dako) or Iba1 (019-19741, Wako) antibodies or direct CFSE visualization.

### 4.10. Coculture Experiment and IFN-*γ* Assay

Spleens from NSG mice were collected and separated in single-cell suspensions. An average of 1.5 × 10^6^ splenocytes were obtained per spleen. Suspensions were stained with an antibody cocktail and then analyzed by flow cytometry for expression of CD11b (Mac-1) versus Gr-1 (Ly-6G) and CD11b versus CD16/32 (FcR*γ*RIII/FcR*γ*RII). Over 80% of splenocytes were positive for CD16/32 (see Figure 7, 1/3 experiments). NK-92 (NK-92^NT^, NK-92^CD16^, or NK-92^CAR^) and splenocytes or the HER2 positive cell line BT474 were cocultured at an effector ratio of 5 : 1 and 2 : 1, respectively, in 96-well round bottom culture plates in RPMI 1640 supplemented with 10% FCS and IL2 (100 IU/mL). After 18 h, supernatants were collected and human IFN-*γ* content was estimated using an ELISA kit (Affymetrix, e-biosciences).

## Abbreviations

CAR: Chimeric antigen receptor
Fc*γ*RIII: Fc receptor
IgG: Low affinity
Fc*ε*RIg: Fc receptor
IgE: High affinity
ADCC: Antibody-dependent cell-mediated cytotoxicity
NK: Natural killer
NSG: NOD scid gamma.
